# Immunotherapy for Cutaneous Squamous Cell Carcinoma: Results and Perspectives

**DOI:** 10.3389/fonc.2021.727027

**Published:** 2022-01-05

**Authors:** Andrea Alberti, Paolo Bossi

**Affiliations:** Medical Oncology, Department of Medical and Surgical Specialties, Radiological Sciences and Public Health University of Brescia, Azienda Socio Sanitaria Territoriale (ASST)-Spedali Civili, Brescia, Italy

**Keywords:** cutaneous squamous cell carcinoma, immunocheck point inhibitors, neoadjuvan, adjuvant, transplantation, future perspective

## Abstract

Although initial surgical excision cures 95% of patients, a minority of cutaneous squamous cell carcinomas (cSCCs) are judged to be unresectable, either locally advanced or with unresectable regional lymph nodes or distant metastases. These patients are offered systemic treatments. Response rate to chemotherapy is relatively low and not durable, as well as the results obtained with epidermal growth factor inhibitors (EGFRi). Like other cutaneous tumors, cSCCs have high immunogenicity, driven by the high mutational burden, the ultraviolet signature, and the overexpressed tumor antigens. Two checkpoint inhibitors, cemiplimab and pembrolizumab, achieved high response rate and survival with fewer toxicities than other available systemic agents. These promising results prompted to investigate new combination strategies of systemic therapy and surgery or radiotherapy. Subgroup analysis showed promising role of immunotherapy to facilitate surgery in locally advanced cSCC and, in a small group of patients, long-term survivals without resection. However, some cSCCs treated with immunotherapy develop either early or late resistance, so new drugs and new combinations are in a clinical study to overcome the mechanism underpinning these resistances. The present review focuses on the progress with immunotherapy to date and on new therapeutic strategies for cSCC.

## Introduction

Cutaneous squamous cell carcinoma (cSCC) is the second most common non-melanoma skin cancer and accounts for 20% of all deaths from skin cancer (1, 2). The estimated incidence of new cSCC cases in the UK is between 15 and 35 per 100,000 people and is increasing (3). The vast majority of patients have a limited disease, so can be successfully managed with a variety of simple procedures, such as cryotherapy and curettage, topical treatments (fluorouracil, imiquimod), or simple surgical excision. When the lesions are more advanced, Mohs micrographic surgery, more extensive surgical resection, or radiotherapy or their combinations are generally sufficient to control the locoregional disease. Only 5% of cSCCs are unresectable, locally advanced, or with non-resectable regional lymph nodes or distant metastases. This quote of patients represents the indication for systemic treatments.

Only limited data are available on the role of systemic chemotherapy in the treatment of advanced cSCC. Cisplatin-based combinations appear to be the most active regimens (4, 5) and have been adapted from those used for SCC occurring at other non-cutaneous sites. Based on non-randomized trials, systemic chemotherapy is able to achieve partial response (PR) in about 34–44% of the cases, with median progression-free survival (PFS) and overall survival (OS) of about 5 and 11 months, respectively (5, 6).

The epidermal growth factor receptor (EGFR) is highly expressed in many epithelial tumors. Although its tumoral expression is inversely correlated with clinical outcome (7), the degree of overexpression does not appear to correlate with the efficacy of EGFR inhibitors (8). In prospective studies on EGFR inhibition with antibodies or small molecules in patients with advanced cSCC, an objective response was reported in 10–31% of patients, and the median time of OS was 11–13 months (8–12). A phase 2 study on cetuximab reported an objective response of 28% and a mean OS of 8.1 months (10). Therefore, advanced cSCC is a life-threatening condition for patients treated with cytotoxic chemotherapy or EGFR inhibitors and is associated with substantial morbidity, quality of life impact, and health care burden. Patients over 65 years of age are more likely than younger patients to require dose reductions in the first cycle of chemotherapy, emphasizing the need for new therapeutic approaches in a predominantly elderly population (13, 14).

## Why Are Cutaneous Squamous Cell Carcinoma So Immunogenic?

Long-term sun exposure leading to DNA damage is postulated to account for the high mutational burden, approximately 45 mutations per megabase (15–18). Furthermore, tumor suppressor genes are most frequently altered, with the UV signature being a key mutational difference (15). Because UV have a relevant role in an early phase of cutaneous cancers pathogenesis, several molecular studies demonstrated that cSCC has a high mutational burden, which likely results in higher levels of tumor neoantigens that may be targets for the immune system. Additionally, the strong link between immunosuppression and the risk of cutaneous squamous cell carcinoma (19) indicates that natural immunosurveillance has a strong role in controlling this tumor type. There are several posited mechanisms for immunosurveillance escape, in cSCC the more relevant being the promotion of an immune-tolerant microenvironment (20). This happens by manipulation of cytokines (increased secretion of IL-6, IL-10, and TGF-beta; consumption of IL-2) that encourages infiltration of Treg cells, myeloid-derived suppressor cells (MDSCs), and other cell types that negatively modulate immune response. These cells can then actively suppress proliferation of CD4+ and CD8+ T lymphocytes that would otherwise recognize tumor antigens (20, 21). cSCC also upregulates the expression of immune checkpoint molecules such as PD ligand 1 (PD-L1) that promote peripheral T cell exhaustion (22).

On the basis of these preclinical data, immune checkpoint inhibitors were tested in advanced cSCC with good results.

## Cemiplimab and Pembrolizumab for Locoregionally Advanced or Metastatic Disease

Cemiplimab and pembrolizumab are fully human monoclonal antibodies belonging to the class of immunoglobulin G4 (IgG4) class, which binds to the PD-1 and blocks its interaction with its ligands PD-L1 and PD-L2. The involvement of PD-1 with its PD-L1 and PD-L2 ligands, which are expressed by antigen-presenting cells and may be expressed by tumor cells and/or other cells in the tumor microenvironment, results in inhibition of the T-cell function, such as proliferation, cytokine secretion, and cytotoxic activity.

Cemiplimab efficacy was investigated in cSCC in two expansion cohorts of phase I multicohort study (n=26 patients) (23, 24), and then in a phase II EMPOWER-CSCC 1 study (n=193 patients) (25–29); both trials had an open-label, multicenter design. The phase I clinical trial demonstrated the safety and the activity of cemiplimab in cSCC. The response rate, as assessed by independent central review, was 50% [95% confidence interval (CI), 30 to 70] with a duration of response that exceeded 6 months in 7 of the 13 responding patients (23). These data have been confirmed across the three parallel treatment groups of phase II clinical trial [i.e., 3 mg/kg once every 2 weeks in Groups 1 (mcSCC) and 2 (lacSCC); 350 mg once every 3 weeks in group 3 (mcSCC)]. Prior systemic treatment for cancer had been received by 33.7% of patients, while 90.2% of patients had previously had surgery for their cancer and 67.9% had received prior radiotherapy (30). An objective response was observed in 49.2, 43.6, and 41.1% of patients, in groups 1, 2, and 3, respectively; with a median time to response of 1.9 months in groups 1 and 2 and 2.1 months in group 3 (25, 26). The response seen with cemiplimab in patients with advanced cSCC appeared to be durable; at the interim analyses, the median duration of response (DOR) and median survival was not reached, after a median follow-up of 16.5, 8.1, and 9.3 months for groups 1, 3, and 2, respectively (25, 26). In mcSCC cohort patients with a DOR ≥12 months was 22 of 29 responders (26) and in lacSCC patients 12 of 34 responders (25). Median OS and PFS were yet to be reached by any treatment group.

Across cSCC, pembrolizumab was investigated in two phase II trials, the KEYNOTE 629 (n= 105 patients) and CARSKIN trial (n=57 patients in expansion cohort); both trials were open-label, single-arm, multicenter design. In KEYNOTE 629, majority of the patients had received one or more prior systemic therapies (87%) or RT (74%) (31). In the entire study population, the objective response rate was 34%, with complete and partial response rates reported in 4 and 31%, respectively. Among the cohort of 36 patients with confirmed disease response, approximately two-thirds (69%) experienced durable responses longer than 6 months. At a median follow-up of approximately 10 months, median PFS was 7 months, and 1-year OS was 60% (31).

In the investigated initiated CARSKIN trial, where only treatment-naïve patients were enrolled, the objective response rate in the entire study population was 42%, with a complete and partial response rate of 7 and 35%, respectively (32). In the expansion cohort, the objective response rate was higher among those with programmed cell death ligand 1 (PD-L1)-positive disease (55%) *versus* PD-L1-negative disease (17%) (P=0.02) (32). In the primary cohort, after a median follow-up of 22.4 months, any of 16 responders experienced a subsequent disease progression. In this cohort, the median PFS and OS were 7 and 25 months, respectively (32) (See **Table 1**).

**Table 1:** Overview of results from Keynote 629, Carskin and EMPOWER trial.

	EMPOWER – CSCC 1 (25–27)			KEYNOTE 629 (31)	CARSKIN (32)
	Group1*	Group2*	Group3*		
Drug	Cemiplimab			Pembrolizumab	Pembrolizumab
Dose	3mg/Kg q2W mCSCC	3mg/Kg q2W laCSCC	350 mg q3W	200 mg q3W	200 mg q3W
Number of pts enrolled	59	78	56	105	57
Prior systemic treatment (%pts)	56	15	36	87	Treatment naive
Median follow-up (months)	16.5	8.1	9.3	11.4	22.4
ORR % (95% CI)	49 (36–63)	44 (32–55)	41 (28–55)	34 (25–44)	42 (29–56)
DCR % (95% CI)	71 (58–82)	79 (69–88)	64 (50–77)	52 (42–62)	60 (46–72)
mDOR (months)	NR	NR	NR	NR	NR
Kaplan–Meier 12-month estimate of DOR, % (95% CI)	89 (69–96.3)	88 (66–95)	NE	66 (NR)	93 (82–100)

* Group 1, 3 mg/kg once every 2 weeks mCSCC; Group 2, 3 mg/kg once every 2 weeks laCSCC; Group 3, 350 mg once every 3 weeks. CSCC, cutaneous squamous cell carcinoma; laCSCC, local advanced CSCC; mCSCC, metastatic CSCC; ORR, overall response rate; DCR, disease control rate; mDOR, median duration of response; NR, not reached; NE, not explain.

Based on these results, even if the lack of major evidence of phase 3 trials and in the absence of a direct comparison between chemotherapy and immunotherapy, FDA and EMA approved cemiplimab and pembrolizumab for the treatment of advanced and metastatic cSCC. NCCN and ESMO guidelines recommend them as first-line therapy.

## Safety and Adverse Events of Immunotherapy in Advanced and Metastatic cSCC

Because of the advanced age and comorbidities in patients with cSCC, safety is one of the most important challenges. Across the above-presented trials, most treatment-related adverse events (TRAEs) were G1 to 2, and only 13–19% were G ≥3. Less than 10% of patients discontinued the treatment because of toxicities, with a low number of treatment-related deaths (25–27, 31, 32). Investigators distinguished “treatment-related adverse events (TRAE)” from “immune-related adverse events (irAE),” the second one linked to the probable immune-pathogenesis. Most immune-mediated AEs were G1 or 2 and non-serious. In KN629, the most frequently reported ones were hypothyroidism (8.8%), pneumonitis (3.8%), hyperthyroidism (3.1%), and severe skin reactions (3.1%), while there were no grade 4 to 5 irAEs (31) (See **Table 2**).

**Table 2:** Overview of adverse events from Keynote 629, Carskin and EMPOWER trial.

	EMPOWER – CSCC 1 (25–27)			KEYNOTE 629 (31)	CARSKIN (32)
	Group1*	Group2*	Group3*		
Drug	Cemiplimab			Pembrolizumab	Pembrolizumab
Dose	3 mg/kg q2W mCSCC	3 mg/kg q2W laCSCC	350 mg q3W	200 mg q3W	200 mg q3W
Any TRAE	58%	78%	64%	67%	71%
TRAE grade ≥3	19%	10%	13%	6%	7%
Treatment-related death	0	1 pt (aspiration pneumonia)	0	1 pt (cranial nerve neuropathy)	1 pt
TRAE-led discontinuation	8%	8%	10%	12%	na
Most frequent TRAE	Fatigue (27%)	Fatigue (28%)	Rash (13%)	Pruritus (14%)	Fatigue (18%)
1^st^			
2^nd^	Arthralgia (8%)	Pruritus (22%)	Fatigue (11%)	Asthenia (13%)	Diarrhea (13%)
3^rd^	Diarrhea (8%)	Diarrhea (17%)	Hypothyroidism (11%)	Fatigue (12%)	Hypothyroidism (13%)

*Group 1, 3 mg/kg once every 2 weeks mCSCC; Group 2, 3 mg/kg once every 2 weeks laCSCC; Group 3, 350 mg once every 3 weeks. CSCC, cutaneous squamous cell carcinoma; laCSCC, local advanced CSCC; mCSCC metastatic CSCC; TRAE, treatment related adverse events.

We may therefore conclude that in advanced and metastatic cSCC, treatment with both cemiplimab and pembrolizumab is safe, with a spectrum of toxicities similar to what observed in other solid tumors. However, because comorbidities in patients with cSCC may be high, any added toxicities impact on patient’s frailty, therefore suggesting the importance of an early recognition and treatment of immune-mediated AEs.

## Immunotherapy in Allogenic Organ Transplantation

It is well-known that the state of immune tolerance induced by broad immunosuppression to prevent allograft rejection leads to an increased risk of the development of cSCC.

Both CTLA-4 and PD-1/PD-L1 play a key role in immunotolerance required for allograft survival (33, 34). In a preclinical study, the injection of anti-CTLA-4 immunoglobulin in the perioperative period led to the acute rejection of liver allograft but did not have any effect on graft survival when it was injected after the establishment of peripheral tolerance (33). On the contrary, the early infusion of anti-PD-1 antibodies prevented the induction of peripheral tolerance, and infusion at a later stage led to the complete loss of allograft (33–35). Although this has not been proved in humans, several reports in literature are warning about the high rates (quite 40%) of allograft rejection in patients with cancer who were treated with an ICI leading to organ failure in 71% of the patients who experienced rejection (36). The majority of graft rejections happened after 1–2 doses of ICIs, although no one has demonstrated that the loss of immunotolerance secondary to ICI is dose- and time-independent.

Accordingly, prospective studies using ICIs in organ-transplanted patients with cancer are needed. The only prospective study reported to date is a small phase I clinical trial (37) testing the safety of nivolumab in four renal transplant recipients with multiple myeloma, head and neck SCC, renal cell carcinoma, and bladder cancer. The patients were required to have no human leukocyte antigen donor-specific antibodies (DSAs). Patients received one, two, three, and nine doses of nivolumab, respectively. None of the patients had a graft rejection, and only one patient (who received nine doses) experienced a partial response. Another phase I trial (38) is open and accruing patients with renal transplants diagnosed with unresectable or metastatic cutaneous carcinoma (melanoma-cSCC-basal cell carcinoma or Merkel cell carcinoma) to receive prednisone, tacrolimus, and nivolumab with the addition of ipilimumab upon the progression of the disease. The primary endpoint of the study is response rate. As of today, because of the high risk of allograft loss and the poor data of clinical benefit, the use of ICI should be discussed with patients clearly before the initiation of treatment, and these patients should be closely monitored for signs of rejection.

## Future Strategies: Adjuvant and Neoadjuvant Immunotherapy in cSCC

Clinical trials in melanoma showed that in the advanced metastatic treatment setting, patients with lower tumor burdens were more likely to experience long-term survival after anti–PD-1 therapy (39, 40). This suggests that postoperative (adjuvant) anti–PD-1 therapy directed against residual micrometastatic disease might prolong RFS and OS. The same impact in survival could be foreseen by anticipating immunotherapy before surgery (neoadjuvant), where using PD-L1 blockade with primary tumor in place could leverage higher levels of endogenous tumor antigen to enhance T-cell priming.

In fact, anti–PD-(L)1 rejuvenates tumor-specific cytotoxic T cells that already reside in the tumor microenvironment (TME), causing their activation, proliferation, and trafficking to micrometastatic deposits. Moreover, having tumor-draining lymph nodes (TDLN) in place could increase the antigen presentation by dendritic cell to T cells. Recently, Yost, Chang, and colleagues showed that after anti–PD-1 therapy of cutaneous squamous cell carcinomas, T cell clonal expansion was due to new clones “appearing” in the TME rather than expansion of clones already in the tumor before initiation of anti–PD-1 therapy; these findings suggest that other clones not present initially in the tumor traffic in it upon anti–PD-1 treatment (41). The melanoma trial by Blank et al. comparing neoadjuvant *versus* adjuvant regimens of anti–PD-1 plus anti–CTLA-4 found a greater expansion of tumor-resident T-cell clones in the peripheral blood of patients enrolled on the neoadjuvant arm (42). These clones persisted in the periphery for weeks after tumor resection (41).

Based on this strong biological rationale and on data from melanoma studies, several ongoing trials are investigating the role of checkpoint inhibitors in neoadjuvant settings for patients with cSCC. They differ from each other at first for inclusion criteria, because they enroll only patients with high-risk tumor, but the risk of recurrence is established by different factors. To date, the most used reference to identify high-risk patients is the American Joint Committee on Cancer staging 8th edition (AJCC-8) (43). High-risk cSCCs are therefore defined according to tumor diameter, lymph node size, the number of positive lymph nodes and their location(s) (ipsilateral, contralateral, bilateral), and extranodal extension. However, the AJCC-8 is relevant only for head-and-neck cSCCs, which might limit its usefulness. Other risk factors are considered by the Brigham and Women’s Hospital (BWH)-staging system (43), when having all of these characteristics: tumor diameter ≥2 cm, tumor invasion beyond subcutaneous fat, perineural invasion ≥0.1 mm and poorly differentiated cSCC, or bone invasion.

After all, the correct selection of patient population in the neoadjuvant setting is an important challenge. Special considerations are centered on risk-benefit expectations in patient populations among which a proportion would be cured by surgery alone and on the other hand the risk of severe and prolonged immune-related adverse effects.

Another point of difference between trials in these settings is the primary endpoint used as surrogate of survival. Disease-free survival is the historical surrogate of overall survival in the adjuvant setting, although differences exist across neoadjuvant immunotherapy clinical studies. This is because the role of response rate as a surrogate of survival in this setting is not well established. Pathologic response criteria for neoadjuvant cancer therapy were first developed in the context of chemotherapy as a parameter portending clinical outcomes. Pathologic complete response (pCR), the most stringent criterion, is defined as the absence of any viable tumor in the definitive surgical resection specimen. To date, pCR and major pathologic response (MPR) defined as describing a treatment effect resulting in ≤10% residual viable tumor are the most commonly used metrics for assessing response to neoadjuvant immunotherapy, although differences exist across clinical studies.

Similar to the precedent established with non-immunologic neoadjuvant therapies, the degree of pathologic response may help assign patients to postsurgical observation or intervention, and ongoing studies will answer this question.

If these new therapeutic strategies would translate into a clinical benefit, new questions will arise regarding the type of surgery and the role of postoperative radiotherapy. At first on the surgical treatment, if an aggressive or cosmetically disfiguring surgery would have been recommended in the absence of neoadjuvant therapy, would the same surgical approach still be required for tumors exhibiting a major response to neoadjuvant treatment? Limited surgical interventions could be used in patients whose on-treatment tumor biopsies show a complete or major pathologic response, for example, as provided in melanoma in an extension cohort of NCT02977052.

## Future Strategies: Immunotherapy Concomitant to Radiation

Another innovative strategy in cSCC therapy is the combination of immunotherapy with radiotherapy. The discovery that radiation-induced damage to tumor tissues and normal tissues in the radiation field can trigger the activation of the immune system *via* well-known damage-signaling cascades, immunogenic cell death, or both has led to a paradigm change in the use of radiotherapy. Preclinical and clinical investigations revealed a complex interplay between radiotherapy, irradiated cells and tissues, and the immune system (44–46); for example, exposure to radiotherapy was shown to upregulate major histocompatibility complex I (MHC I) expression in tumor cells, modulate immunosuppressive barriers in the tumor microenvironment, activate restrictive tumor vessels, trigger the recruitment of immune effector cells to the local tumor, and even elicit systemic tumor-specific immune responses (47, 48). The efficacy of synergy between radiotherapy and checkpoint inhibitors in squamous cell carcinoma of the skin is being studied in the UNSCARRed trial: a single-arm, interventional study combining avelumab with radical radiotherapy, accrual is ongoing.

## New Immunotherapy Drugs and Combinations

Increased understanding of the underlying immunologic mechanisms is leading to the identification of several additional potential targets to unleash the immune system and control malignancy. These approaches include new checkpoint inhibitors, agonist of costimulatory receptors, manipulation of T cells, oncolytic viruses, cytokines, and vaccines. In addition to implement the response to immunotherapy and increase survival, new pharmacological combinations are under investigation.

### New Checkpoint Inhibitors and Combinations

Data on anti-CTLA4 (e.g., Ipilimumab or tremelimumab) for cSCC are limited; their association with anti-PD1 is still in study in the neoadjuvant setting (NCT04620200) and in allograft patients (NCT03816332) (38).

TIGIT is another inhibitory receptor co-expressed with PD-1 on tumor antigen-specific CD8+ T cells and CD8+ tumor-infiltrating lymphocytes (TILs). It is highly expressed by Tregs in peripheral blood mononuclear cells of healthy donors and patients with cancer and further upregulated in the TME (49). Promising data on anti-TIGIT monoclonal antibody Tiragolumab efficacy in NSCLC had been presented at ASCO virtual meeting 2020 (50). As far as we know, there are no trials ongoing with this drug in cSCC. CD47 is another promising target. It is upregulated in essentially every cancer type to inhibit innate immune cells from phagocytosing the tumor cells. A humanized anti-CD47 monoclonal antibody demonstrated promising results in non-Hodgkin lymphoma (51), and the evaluation of the activity in cSCC is ongoing (NCT04502888).

Another therapeutic strategy, formulated to increase the response rate of immunotherapy and to overcome mechanisms of resistance to progression, is the addition of an anti-EGFR agent. The hypothesis of I-TACKLE (NCT03666325) trial is that the adjunct of an anti EGFR agent as cetuximab could reverse the primary and secondary resistance to pembrolizumab, with a synergistic effect able to counteract pathway redundancy (i.e., the presence of several concurrent pathways which need to be addressed together) and boosting T-cell priming. Enrollment is underway, and the results of a first analysis are expected soon. Another trial with avelumab concurrently with cetuximab is currently ongoing (NCT03944941).

Checkpoint inhibitors and modulators of DNA damage response (DDR) is another pharmacological association being studied in several neoplasms. PARPi-mediated catastrophic DNA damage is a favorable factor for ICI therapy, and the relationship between tumor mutation burden and efficacy of ICI has been confirmed in previous studies (52). Apart from tumor mutation burden, DDR-mediated immune responses collaborate with ICI, which remodel tumor immune microenvironment and boost the cancer immunity cycle (53). In this way, PARPi-mediated acute inflammation remodels tumor immune microenvironment and drives a systemic Th1-skewing immune response. In cSCC, a trial is ongoing with pembrolizumab plus abexinostat (NCT03590054), a pan-histone deacetylase inhibitor and inhibitor of RAD51, which is involved in repairing DNA double-strand breaks.

### Agonists of Costimulatory Receptors

Multiple costimulatory receptors are involved in the immune response to tumors and hence are potential targets for cancer immunotherapy. Inducible T-cell costimulator (ICOS), CD40, CD28 agonists are only some examples of costimulatory receptors, studied in preclinical animal models and some of them also in early phases of clinical development (54). CD40 has been identified as an interesting immunotherapy target in human cancers by virtue of its ability to stimulate helper T-cell immune response and macrophage differentiation (55). CD40 ligand (CD40L) gene therapy has been shown to increase tumor-infiltrating T cells *in vivo* and demonstrated an oncolytic effect. SL-172154 is an engineered monoclonal antibody that consists of Sirpα linked to CD40L, providing checkpoint blockade (CD47 axis) and potent costimulation (CD40 axis). By blocking this signal through Sirpα binding, the surface of the tumor cells is coated with the drug, allowing the CD40L side to bind to CD40 on APCs, which will lead to enhanced antigen presentation to CD8+ and CD4+ T lymphocytes and tumor cell phagocytosis. The trial with SL-172154 in cSCC is ongoing (NCT04502888).

### Cytokines

Initial approaches to immunotherapy harnessed the numerous downstream effects of cytokines. IL2 and INFa demonstrate mild efficacy in melanoma and renal cell carcinoma. Other interleukin analogs or interleukin receptor agonists have been studied in the preclinical setting, with poor results. Today promising results are expected by the combination between atezolizumab and NT-I7 (recombinant human IL-7-hybrid Fc), which acts through IL-7 receptor (IL-7R), which is expressed on naïve and memory CD4+ and CD8+ T cells. Thus, IL-7 promotes proliferation, maintenance, and functionality of these key T-cell subsets mediating immune responses. The trial with this association in cSCC is ongoing (NCT03901573).

### Oncolytic Viruses

Oncolytic viruses mediate antitumor effects in several ways. Viruses can be engineered to efficiently infect cancer cells preferentially over normal cells, to promote the presentation of tumor-associated antigens, to activate signals that promote a less immune-tolerant tumor microenvironment, and to serve as transduction vehicles for the expression of immune-modulatory cytokines (56). Injection of oncolytic viruses may synergize with checkpoint inhibitors by increasing CD8+ T cell infiltration and IFN gamma signaling as well as upregulating PD-L1 in the microenvironment (57).

RP1 is an oncolytic HSV that encodes a fusogenic GALV-GP R- protein and granulocyte-macrophage colony-stimulating factor (GM-CSF). RP1 demonstrated tolerable safety and tumor regression alone and with nivolumab in patients with several tumor types, including cSCC (57). Another promising oncolytic virus is Talimogene laherparepvec (TVEC). It is again an HSV-1, modified to lose the neurovirulence and include the capacity to express GM-CSF. This allows for preferential replication within tumor cells resulting in cell lysis. Additionally, the release of virally derived GM-CSF along with antigens derived from ruptured tumor cells can induce a systemic tumor-specific immune response which may lead to regression of distant uninjected lesions (58).

### Vaccines

Many attempts have been made to harness the adaptive immune recognition of a cancer-related antigen to effect antitumor responses. There are many methods of vaccination, and the choice of antigen, schedules of administration, and adjuvants can influence an adaptive immune response. Antigen choices range from simple peptides, which are easy to administer but affect a narrow antigen spectrum and are often restricted by specific HLA class 1, to whole-cell preparations that offer a broader range of antigens but are more costly and time-consuming to prepare (59). Given the increasing understanding of the importance of immune recognition of not only multiple tumor antigens but specific ones for each patient, current studies to develop therapeutic vaccines are beginning to explore the use of individualized pooled antigens. Several efforts on these strategies are being made in cSCC.

Other drugs with alternative mechanisms of action, external to the immune synapse, are under investigation. Among the molecular ones suitable to stimulate antitumor immune effects, a toll-like receptor 9 (TLR9) agonist, CMP-001, activates plasmacytoid dendritic cells (pDCs) and triggers interferon alpha (IFNα) release. These lead to a cascade of antitumor immune effects. Ongoing trials are testing CMP-001 in combination with checkpoint inhibitors in several solid tumors, also in cSCC (NCT03684785).

## Conclusions

The treatment of locally advanced metastatic cSCC remains a challenge. Chemotherapy may achieve response rata in about one-third of the patients, as targeted agents against EGFR (4, 5, 10); however, the main limitations of these approaches are the relatively short duration of response and the adverse events, often not compatible with the frailty of the patients. In contrast, immune checkpoint inhibitors showed a high response rate, a long duration of response, and a better toxicity profile (25–27, 31, 32). This has broadened the therapeutic armamentarium to be offered to cSCC patients and the possibility to treat also elderly patients with comorbidities otherwise not amenable to systemic treatments. However, new questions arise from this new approach. First of all, the search for predictive factors of response to treatment remains an unresolved challenge, with no molecular or clinical factor able to identify patients with a greater possibility of obtaining a benefit from immunotherapy. Moreover, it is not clear up to now how long to continue the treatment in patients who achieve a complete response with immune checkpoint inhibitors. The role of immunotherapy as adjuvant or neoadjuvant treatment is being investigated in ongoing clinical trials, thus representing a new frontier for multidisciplinary approaches. Behind this, if immunotherapy has proven to be an effective strategy, new drug combinations with novel mechanisms of actions are being investigated to improve the results till now considered and to overcome the resistance mechanisms.

## Author Contributions

PB contributed to conception of the study. AA wrote the first draft of the manuscript. PB and AA contributed to manuscript revision, read, and approved the submitted version.

## Conflict of Interest

PB declares advisory board or conference honoraria from Merck, Sanofi-Regeneron, Merck Sharp & Dohme, Sun Pharma, Angelini, Molteni, Bristol-Myers Squibb, GSK, Nestlè.

The remaining author declares that the research was conducted in the absence of any commercial or financial relationships that could be construed as a potential conflict of interest.

## Publisher’s Note

All claims expressed in this article are solely those of the authors and do not necessarily represent those of their affiliated organizations, or those of the publisher, the editors and the reviewers. Any product that may be evaluated in this article, or claim that may be made by its manufacturer, is not guaranteed or endorsed by the publisher.
